# Both STING and MAVS Fish Orthologs Contribute to the Induction of Interferon Mediated by RIG-I

**DOI:** 10.1371/journal.pone.0047737

**Published:** 2012-10-16

**Authors:** Stéphane Biacchesi, Emilie Mérour, Annie Lamoureux, Julie Bernard, Michel Brémont

**Affiliations:** Unité de Virologie et d’Immunologie Moléculaires, INRA, CRJ, Jouy-en-Josas, France; McMaster University, Canada

## Abstract

Viral infections are detected in most cases by the host innate immune system through pattern-recognition receptors (PRR), the sensors for pathogen-associated molecular patterns (PAMPs), which induce the production of cytokines, such as type I interferons (IFN). Recent identification in mammalian and teleost fish of cytoplasmic viral RNA sensors, RIG-I-like receptors (RLRs), and their mitochondrial adaptor: the mitochondrial antiviral signaling (MAVS) protein, also called IPS-1, highlight their important role in the induction of IFN at the early stage of a virus infection. More recently, an endoplasmic reticulum (ER) adaptor: the stimulator of interferon genes (STING) protein, also called MITA, ERIS and MPYS, has been shown to play a pivotal role in response to both non-self-cytosolic RNA and dsDNA. In this study, we cloned STING cDNAs from zebrafish and showed that it was an ortholog to mammalian STING. We demonstrated that overexpression of this ER protein in fish cells led to a constitutive induction of IFN and interferon-stimulated genes (ISGs). STING-overexpressing cells were almost fully protected against RNA virus infection with a strong inhibition of both DNA and RNA virus replication. In addition, we found that together with MAVS, STING was an important player in the RIG-I IFN-inducing pathway. This report provides the demonstration that teleost fish possess a functional RLR pathway in which MAVS and STING are downstream signaling molecules of RIG-I. The Sequences presented in this article have been submitted to GenBank under accession numbers: Zebrafish STING (HE856619); EPC STING (HE856620); EPC IRF3 (HE856621); EPC IFN promoter (HE856618).

## Introduction

The innate immune system recognizes pathogen components through pattern-recognition receptors (PRRs) that are specific for conserved pathogen-associated molecular patterns (PAMPs), such as pathogen-derived nucleic acids and viral proteins. The recognition of PAMPs ligands leads to the activation of multiple signaling cascades that induce production of interferons (IFN) and other cytokines. This innate immune response is essential for successful pathogen early elimination and is also crucial in the induction of the specific adaptive immune response. Among PRRs, retinoic acid-inducible gene-I (RIG-I)-like receptors (RLRs) play a key role in sensing non-self RNA in the cell cytosol and are essential in the early induction of type I IFN [1]. The RLR family comprises three helicases, RIG-I [2], melanoma differentiation-associated gene 5 (MDA5) [3], [4], [5], and laboratory of genetics and physiology 2 (LGP2) [6], [7], all of which containing a DExD/H-box RNA helicase domain. Only, RIG-I and MDA5 possess two N-terminal caspase-recruitment domains (CARDs). Overexpression of the CARD alone is sufficient to drive antiviral signaling resulting in IFN production, showing that this domain is responsible for signaling [6]. RIG-I binds preferentially, but not exclusively, to ssRNA that are phosphorylated at the 5′-end, whereas MDA5 recognizes long dsRNA that do not necessitate 5′-phosphorylation [8]. The ligand preferences of these proteins result in the detection of a wide variety of positive- and negative-stranded RNA viruses, and indirectly some DNA viruses involving DNA-dependent RNA polymerase III [9], [10], [11], [12]. In uninfected cells, RIG-I is maintained in an inactive conformation by a C-terminal repressor domain (RD) which masks the CARDs. Following recognition of non-self-cytosolic RNA, the CARDS are released from RD repression and the active RIG-I conformation can bind via homotypic CARD-CARD interactions with the downstream adaptor protein, the mitochondrial antiviral signaling (MAVS) protein (also known as IPS-1, IFN-β promoter stimulator 1; VISA, virus-induced signaling adaptor; and Cardif, CARD adaptor inducing IFN-β), located on the outer mitochondrial membrane [13], [14], [15], [16]. The mitochondrial location of MAVS is essential to trigger further signaling events [14], [16]. The activation of MAVS leads to the recruitment of several downstream signaling molecules and the activation of transcription factors IRF3/7 and NF-κB which drive the expression of the type-I IFN and inflammatory cytokines [12]. Deficiency in both RIG-I and MAVS expressions impairs antiviral response and increases susceptibility to RNA virus infection *in vitro* and *in vivo* [5], [17], [18], [19].

Structurally, MAVS contains an N-terminal CARD for interaction with RIG-I, a central prolin-rich domain involved in protein-protein interactions and a C-terminal transmembrane domain (TM) that inserts MAVS into the outer mitochondrial membrane. MAVS was shown to interact with a wide range of partners which either positively or negatively regulate various pathways and processes: antiviral response, inflammation, apoptosis, autophagy, mitochondrial and peroxisomal dynamics, proteasome degradation and posttranslational modifications such as ubiquitination and phosphorylation (for review [20]). Among these partners, the recently identified stimulator of interferon genes (STING) protein (also called MITA, mediator of IRF3 activation; ERIS, endoplasmic reticulum interferon stimulator; MPYS, N-terminal methionine-proline-tyrosine-serine protein; whereas its gene name is *Tmem173*, transmembrane protein 173) was shown to play a pivotal role in response to both non-self-cytosolic RNA and dsDNA [21], [22], [23], [24], [25]. STING contains multi-putative TM in the N-terminal region and was found to predominantly localize in the endoplasmic reticulum (ER). Overexpression of STING significantly induces type I IFN and IFN-stimulated genes (ISGs), and conversely, deficiency in STING expression impairs the antiviral response and increases susceptibility to DNA and RNA viruses, certain intracellular bacteria and even parasite [22], [26], [27], [28], [29], [30]. In contrast to DNA viruses for which STING is essential to build a strong immune response, STING is not crucial but significantly facilitates innate immune responses against negative-stranded RNA viruses including vesicular stomatitis virus (VSV) and Sendai virus [21], [25]. STING-knockout mice were found highly sensitive to lethal VSV infection [22]. Thus, STING is necessary for efficient, early induction of type I IFN production mediated by RIG-I and is required for protection against negative-stranded RNA virus infection. In contrast, STING does not appear to be necessary for synthetic dsRNA [poly:(IC)] and encephalomyocarditis virus (EMCV, a positive-stranded RNA virus) to induce IFN suggesting that STING may not influence MDA5 function. Immunoprecipitation experiments demonstrated that STING interacts with both RIG-I and MAVS. STING also interacts with IRF3 and recruits the kinase TBK1 (TANK-binding kinase 1), leading to IRF3 activation by phosphorylation and expression of type I IFN [21], [25], [31]. Thus, STING is a key component of RIG-I pathway which is involved downstream or in parallel to MAVS in response to specific stimuli. Finally, STING was shown to be targeted by certain viral proteins, such as dengue virus NS4B protein and coronavirus papain-like proteases, to block downstream signaling [22], [32].

Recently, several studies have demonstrated that teleost fish possess a functional RLR pathway that includes orthologs of human RIG-I, MDA5 and LGP2 [33], [34], [35], [36], [37], [38], [39] and several of the downstream signaling molecules, such as MAVS, TBK1 and IRF3/7 [33], [38], [40], [41], [42], [43], [44], [45], [46]. Overexpression of either full-length RIG-I molecule or the N-terminal CARD domain of RIG-I leads to a strong induction of both IFN and ISGs, conferring on fish cells high protection against RNA virus infection [33], [38]. Similarly, overexpression of the mitochondrial RIG-I adaptor molecule, MAVS, induces constitutive expression of IFN and ISGs leading to the establishment of a strong antiviral state against both RNA and DNA viruses [33], [42], [44]. These observations suggest that IFN-inducing RIG-I-like pathway is highly conserved between teleost fish and mammals. Recently, Sun and colleagues have shown that goldfish (*Carassius auratus*) also possesses an ortholog of STING which contributes to the antiviral response mediated by RIG-I pathway [38]. They provide evidence that fish have a conserved RIG-I-STING-TBK1-IRF3-IFN signaling cascade, but the role of MAVS, the adaptor molecule of RIG-I, was not addressed. In the present study, we cloned STING-like cDNAs from two additional fish species (zebrafish; *Danio rerio* and fathead minnow; *Pimephales promelas*) and showed that they were true orthologs to mammalian STING. We demonstrated that overexpression of these ER proteins in fish cells led to a constitutive induction of IFN and ISGs, and conferred a strong antiviral state against both RNA and DNA viruses. In addition, we showed that STING and MAVS were key components of the RIG-I pathway. These data demonstrate that a functional RLR-based induction pathway of IFN is conserved in vertebrates in which MAVS and STING are downstream signaling molecules of RIG-I.

## Materials and Methods

### Cells and Viruses

EPC cells were maintained in GMEM/HEPES 25 mM medium supplemented with 10% fetal bovine serum and 2 mM L-glutamine. The EPC cells (*Epithelioma Papulosum Cyprini*) were originally described as isolated from common carp (*Cyprinus carpio*) and was subsequently found to be from fathead minnow (*Pimephales promelas*) [33], [47]. Recombinant viral hemorrhagic septicemia viruses (rVHSV of wild-type phenotype and rVHSV-Tomato expressing the tdTomato fluorescent protein (Clontech) were previously described and are derived from the hypervirulent VHSV 23–75 French strain [48], [49]) and epizootic haematopoietic necrosis virus (EHNV) Australian strain [50] were propagated in monolayer cultures of EPC cells either at 14°C (rVHSV) or at 25°C (EHNV) in presence of 2% fetal bovine serum. Virus titers were determined by plaque assay on EPC cells under agarose overlay (0.35% in GMEM/HEPES medium). Two to four days postinfection, cell monolayers were fixed with 10% formol and stained with crystal violet.

### Molecular Cloning and Sequencing of Zebrafish STING and Fathead Minnow STING, IRF3 and IFN Promoter Core

Entire or partial STING sequences were obtained using BLAST analysis of zebrafish (*Danio rerio*, XM_694397) nucleotide collection or salmon (*Salmo salar*, GE786872), carp (*Cyprinus carpio*, EC394745) and weather loach (*Misgurnus anguillicandatus*, BJ827384) expressed sequence tags (ESTs) with homology to mammalian STING gene sequences. Total RNA from ZF4-7 (zebrafish, ATCC #CRL-2050) and EPC cells were extracted using RNeasy kit (QIAGEN) according to the manufacturer’s instructions. The RNA was used to generate full-length cDNAs using the SMART RACE cDNA amplification kit (BD Clontech) with universal primers provided by the manufacturer and gene-specific primers designed from the ESTs sequences. PCR amplifications were performed using the Advantage 2 PCR kit (BD Clontech) and following the manufacturer’s instructions. RT-PCR products were purified with QIAquick PCR purification kit (QIAGEN) and fully sequenced by primer-walking. Specific primers were then designed and used to amplify full-length open reading frames (ORF) of STING (zebrafish, 5-EcoRI-zfSTING: ccgaattcatgtctgtgatgggagaagacgctctcgtcc and 3-XhoI-zfSTING: ggctcgagttagttttgtttcattgcgctagatggg; EPC, 5-EcoRI-epcSTING ccgaattcatgtgtggtgtgatcggagaggacg and 3-XhoI-epcSTING: ggctcgagctaataatcagtagtctccactgg). cDNAs were cloned into the eukaryotic expression vectors pcDNA1.1/Amp (Invitrogen) and peGFP-C1 (Clontech). The cDNA corresponding to the C-terminal part of STING (amino acids 177 to 398) was amplified and cloned into the pcDNA1 vector (using 5-EcoRI-zfSTING Cter: ccgaattcatgagagaatactctagaaggg and 3-XhoI-zfSTING). The sequence of each cloned gene was confirmed by nucleotide sequencing. The specific primers used to amplify by RT-PCR the IRF3 gene expressed by EPC cells were designed from the sequence of the zebrafish (*Danio rerio*) IRF3 gene (GenBank NM_001143904; 5-BglI-IRF3: ccagatctatgactcaagcaaaaccgctg and 3-XhoI-IRF3: ggctcgagttagcagagctccatcatttgc). The cDNA corresponding to the C-terminal part of IRF3 (amino acids 115 to 449) was then amplified (using 5-BglI-IRF3 Cter: ccagatctatgtcggaagggtctcaagagactg and 3-XhoI-IRF3) and cloned into the pcDNA1 vector. Finally, the specific primers used to amplify by PCR the promoter core of EPC cell IFN1 gene were designed from the sequence of the zebrafish IFNФ1 gene promoter (GenBank DQ855952; 5-KpnI-IFNpro: aaggtaccgaccttgaaatactttggaatcaggtaattattttg) and from the sequence of the EPC IFN mRNA (GenBank FN178457; 3-KpnI-IFNpro: aaggtaccgcacaaacatatacgtccacatttgagttttcat). EPC cell genomic DNA was purified using Wizard Genomic DNA Purification kit (Promega). The PCR product was then fully sequenced and a fragment KpnI/XhoI was cloned upstream the firefly luciferase (LUC) gene in the pBlueScript II SK- vector (GenBank X52324), leading to the final construct pIFNpro-LUC. Each plasmid construct used in the present study is briefly described in the Table S1.

### STING Sequence Analysis

The multiple alignments of STING sequences were generated using the software alignX from VectorNTI Advance 11 (Invitrogen) and the TM predictions using the TMpred server (http://www.ch.embnet.org/software/TMPRED_form.html). The Neighbor-joining (NJ) phylogenetic tree of STING was calculated by MEGA5 software [51] based on a multiple alignment (using ClustalW) of full-length and partial STING amino-acid sequences from fish and other vertebrates and a 1000-boostrap was performed. The conserved synteny around the *TMEM173* gene in zebrafish, mouse and human was performed based on the data from the genome assemblies available at NCBI (http://www.ncbi.nlm.nih.gov/).

### Transfection, Infection, Fluorescence Microscopy and Luciferase Activity Assay

EPC cells were plated into 6-well plates at a concentration of 5×10^6^ cells per well 24 h prior to transfection by electroporation (Amaxa Biosystems; Lonza). Cells were trypsinized and resuspended in 100 µl of solution T. Cells were then mixed with 2 µg of plasmid DNA and electroporated using the program T-020. Finally, cells were split equally into two wells of 12-well plates or four wells of 24-well plates. Cell monolayers were washed 24 h posttransfection and a well of each transfection was infected with 0.01 to 1 PFU/cell of VHSV or EHNV 48 h posttransfection. After one hour of adsorption, the inoculum was removed, the cell monolayer washed twice and medium samples (0.5 ml of the 2-ml overlay) were taken (0 time point) and replaced by an equivalent volume of fresh medium. 24 h, 48 h, 72 h or 96 h postinfection, supernatant aliquots were harvested and analyzed later by plaque assay. Cells monolayers were either stained with crystal violet or subjected to total RNA extraction using RNeasy kit (QIAGEN). For immunofluorescence microscopy, specific subcellular compartment of eGFP, eGFP-STING and eGFP-MAVS transfected cells were *in vivo* stained by co-transfecting 1 µg of plasmid DNA encoding the red fluorescent protein fused to an endoplasmic reticulum retention signal (RFP-KDEL) or using 400 nM of MitoTracker Red 580 FM (Invitrogen) for the mitochondria. Cell monolayers were then visualized with a UV-light microscope (Carl Zeiss). The observation of cell monolayers co-expressing eGFP-STING and Cherry-MAVS was performed using a LSM510 confocal microscope (Zeiss). For IFN promoter reporter assays, EPC cells (5×10^6^ cells per well of 6-well plate) transfected by electroporation (see above) with pIFNpro-LUC and various plasmid DNA constructs were seeded into two wells of 12-well plates and incubated at 20°C. At 24 h and 48 h posttransfection, cell lysates were performed using 300 µl of cell culture lysis reagent per well according to the manufacturer’s instructions (luciferase reporter assay system - Promega). eGFP expression from peGFP or pRIG-I Nter-eGFP was measured from 75 µl of cell lysates on a Tecan infinite M200 Pro reader using an excitation wavelength of 480 nm and an emission wavelength of 510 nm. Luciferase activity was then measured by adding 75 µl of luciferase assay reagent on a Tecan infinite M200 Pro reader. Values of luciferase activities were normalized to the levels of eGFP fluorescence. The fold-induction was calculated as the ratio of stimulated versus unstimulated (pcDNA alone) samples. All data shown are representatives of at least three independent experiments.

### RNA Isolation and qRT-PCR Analysis

Total RNA was extracted using RNeasy kit (QIAGEN) from EPC cells transfected with eukaryotic expression vectors encoding STING, MAVS or an empty vector as a control and infected or not with 1 PFU/cell of VHSV at 24 h postinfection. Real-time RT-PCR was performed using one-step reverse transcription and real-time quantitative PCR with RNase-free DNAseI-treated RNA (EXPRESS One-Step SYBR GreenER Kits; Invitrogen Life Technologies) and a MasterCycler Realplex (Eppendorf), according to the manufacturer’s instructions. All reactions were performed in duplicate from three independent experiments. Data analysis was performed as described in the ABI PRISM 7700 sequence detection bulletin no. 2 from Applied Biosystems (http://www3.appliedbiosystems.com/cms/groups/mcb_support/documents/generaldocuments/cms_040980.pdf). Briefly, the relative fold of induction of the gene of interest, normalized to an endogenous reference (β-actin) and relative to a calibrator (pcDNA condition), is given by: 2^−ΔΔCT^. Oligonucleotides used for real-time RT-PCR were designed from β-actin, Viperin, IFN and RIG-I as previously published [33].

## Results

### The Interferon Stimulator Protein STING has a Counterpart in Teleost Fish

**Figure 1:**
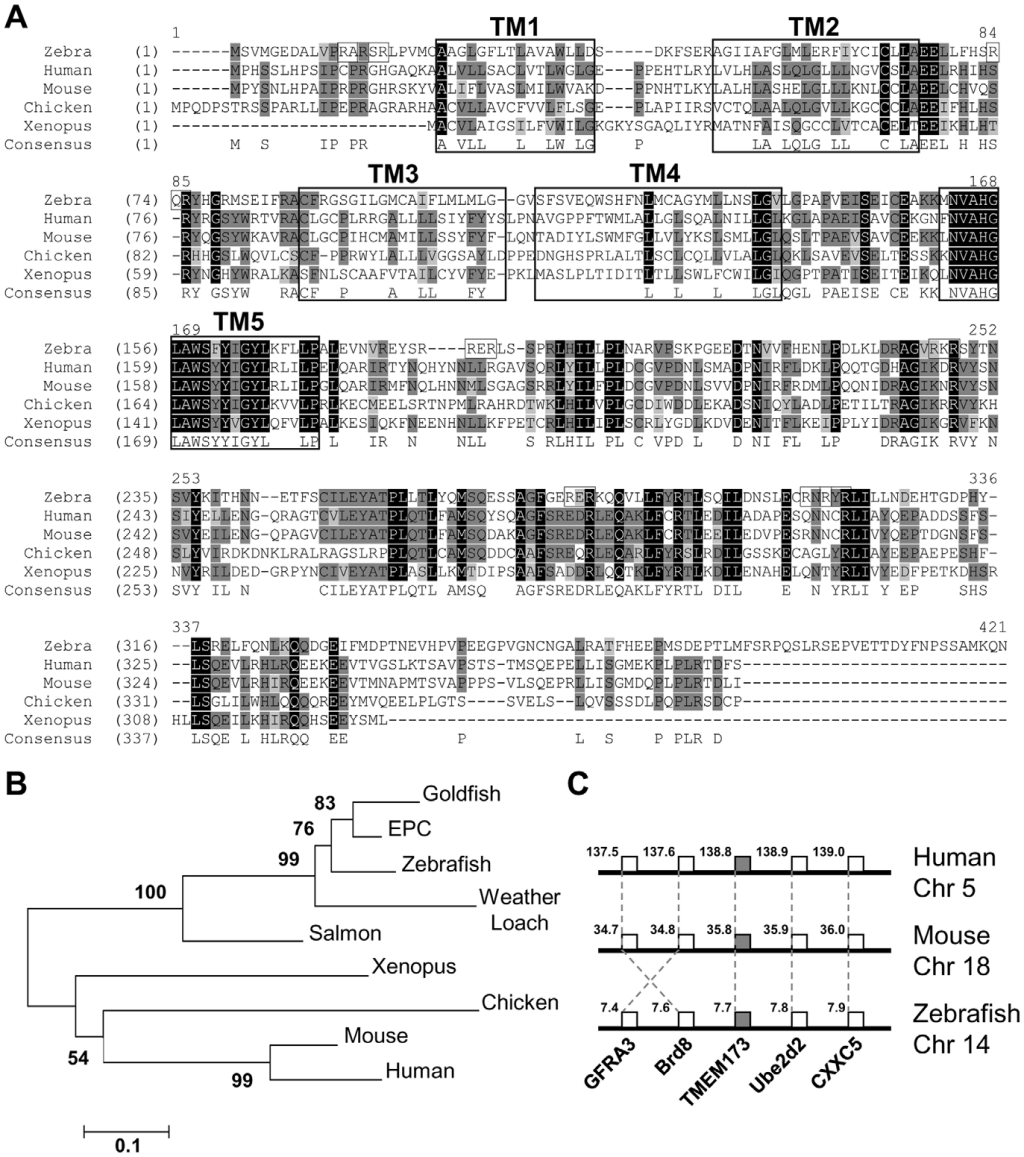
STING sequences through vertebrate evolution. **A.** **Multiple alignment of STING sequences from zebrafish and other vertebrates.** Identical positions are boxed in black, conservative positions in grey and block of similar residues in light grey. The residues of the 5 putative transmembrane (TM) regions and the putative RXR ER retention motifs found in zebrafish STING sequence are boxed. Zebrafish (HE856619, this study), *Danio rerio*; human (NP_938023), *Homo sapiens*; mouse (NP_082537), *Mus musculus*; chicken (E1C7U0), *Gallus gallus*; xenopus (NP_001106445), *Xenopus tropicalis*. **B.** **NJ phylogenetic tree of vertebrate STING.** The tree was based on multiple alignments of full-length and partial STING amino-acid sequences from fish and other vertebrates. The tree is drawn to scale. Full-length sequence accession numbers are the following: EPC (HE856620, this study), *Pimephales promelas*; goldfish (JF970229), *Carassius auratus*, the others are listed above and partial STING amino-acid sequences were deduced from the following EST sequences: salmon (GE786872), *Salmo salar*; weather loach (BJ827384), *Misgurnus anguillicandatus*. **C.** **Conserved synteny around the** ***TMEM173*** **gene in zebrafish, mouse and human.** The location of the different markers and the chromosomes involved are indicated for the different species.

To get insight into the interferon inducing pathway in fish, we cloned the full-length STING-related cDNA of zebrafish and EPC cells. A sequence similar to the human STING was identified in zebrafish genome sequence databases (XP_699489) and in a carp skin EST library (EC394745). Specific primers were designed and used to amplify a cDNA molecule from the zebrafish cell line ZF4-7 and EPC cells. The zebrafish cDNA is of 1197 nt in length and encodes an ORF of 398 aa (GenBank accession no. HE856619) with five predicted transmembrane (TM) regions (Figure 1A). The STING protein from ZF4-7 cells is 99% identical to the sequence available in the bank with 3 amino acid differences: K113E, E205G and Q356R. This sequence was subjected to multiple alignments with the human, mouse, chicken and xenopus STING, showing that the sequence from zebrafish displays only a weak similarity with its counterparts (38–42%), except for certain motifs that are highly conserved among species, such as TM5 which was recently shown to be cytosolic and a dimerization domain [52]. Several putative motifs (RXR) found in resident ER proteins are present along the zebrafish STING sequence (Figure 1A). The EPC cDNA is of 1164 nt in length and encodes an ORF of 387 aa (GenBank accession no. HE856620) displaying as expected a high identity with that of zebrafish (71%).

To get insight into the STING evolution, we searched for STING in the zebrafish genome. We could find only one *sting* gene on chromosome 14. We could also identify partial STING-related sequences in salmon, and in weather loach, suggesting that a STING-related molecule is present in teleost fish, as recently found by Sun and colleagues in goldfish (*Carrassius auratus* L.) [38]. A phylogenetic analysis suggested that all these sequences constitute true orthologs (Figure 1B). To confirm that zebrafish sequence corresponds to the orthologs of the antiviral mammalian *sting*, we search for conserved synteny involving *sting* and other markers in the neighborhood. We could identify four markers that are conservatively found in the genomic region where *sting* is located in zebrafish and mammals (Figure 1C), reinforcing the idea that they are all true orthologs. From the protein structure, phylogenetic analysis and conserved synteny, we therefore conclude that teleost fish possess a *sting* gene that is orthologous to the molecule involved in the regulation of IFN expression in human and in mouse.

### Overexpression of STING Induces a Strong Antiviral State in Fish Cells against both RNA and DNA Viruses

**Figure 2:**
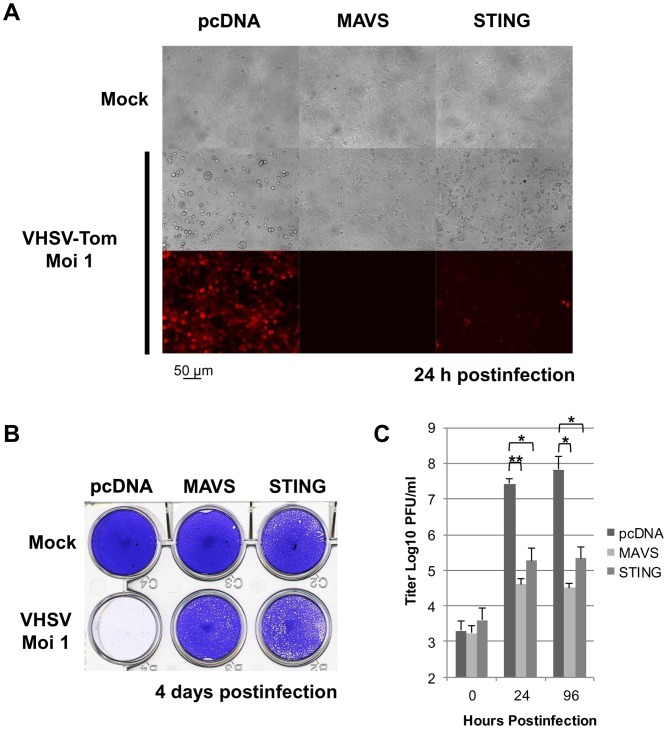
Zebrafish STING is a strong antiviral protein. EPC cells were transfected with 2 µg of pcDNA-STING, and pcDNA-MAVS or an empty vector (pcDNA) as positive and negative controls, respectively. At 48 h posttransfection, EPC cells were infected with a recombinant rVHSV-Tom expressing the tdTomato fluorescent protein at an MOI of 1 and incubated at 15°C. Cell monolayers were visualized under a UV-visible light microscope at 24 h postinfection (A) and then stained with crystal violet 4 days postinfection (B). The culture supernatants from cells infected with rVHSV-Tom were collected at 0, 24 and 96 h postinfection and the viral titer was determined by plaque assay on EPC cells (C). Each time point was represented by three independent experiments, and each virus titration was done in duplicate. Means are shown. The standard errors were calculated and the error bars are shown. Asterisks indicate significant difference (*p<0.01 and **p<0.001) as determined by Student’s *t* test.

Since human and mouse STING overexpressions are sufficient to delay the replication of the vesicular stomatitis virus (VSV) [21], a RNA virus belonging to the Rhabdoviridae family, we examined whether the zebrafish STING could mediate a similar effect in fish cells. Fish EPC cells were transfected with 2 µg of pcDNA-STING. As negative control, EPC cells were transfected with the same amount of an empty vector (pcDNA) or as positive control, an expression vector expressing MAVS from zebrafish which was previously shown to induce antiviral immunity [33]. Two days posttransfection, the cells were infected with a recombinant fish rhabdovirus expressing the Tomato reporter gene, rVHSV-Tom [49], at a MOI of 1 and incubated at 15°C. EPC cells transfected with an empty vector and infected by rVHSV-Tom led to the infection of the entire cell monolayer 24 hours postinfection as visualized with a fluorescent microscope (Figure 2A) and a complete cytopathic effect (CPE) in 3 to 4 days (Figure 2B). In contrast, transfection of these cells with a vector encoding the zebrafish STING protected them against rVHSV-Tom infection (Figure 2A and 2B). Nevertheless, in contrast to the cell monolayer overexpressing the antiviral MAVS protein, VHSV-positive cells could be observed in STING-transfected cell monolayer leading to a limited CPE. Measurement of the viral titer showed that overexpression of zebrafish STING decreased the viral titer by 444-fold as compared to that in control cells (from 7.1×10^7^ to 1.6×10^5^ PFU/ml at 4 days postinfection; Figure 2C). Moreover, the viral titer only increased by 40-fold in 4 days in STING-expressing cells compared to 35,500-fold for the control cells. Similar results could be observed with EPC STING (data not shown). These results show that STING is a cellular antiviral protein whose overexpression induces a strong antiviral immunity in fish.

**Figure 3:**
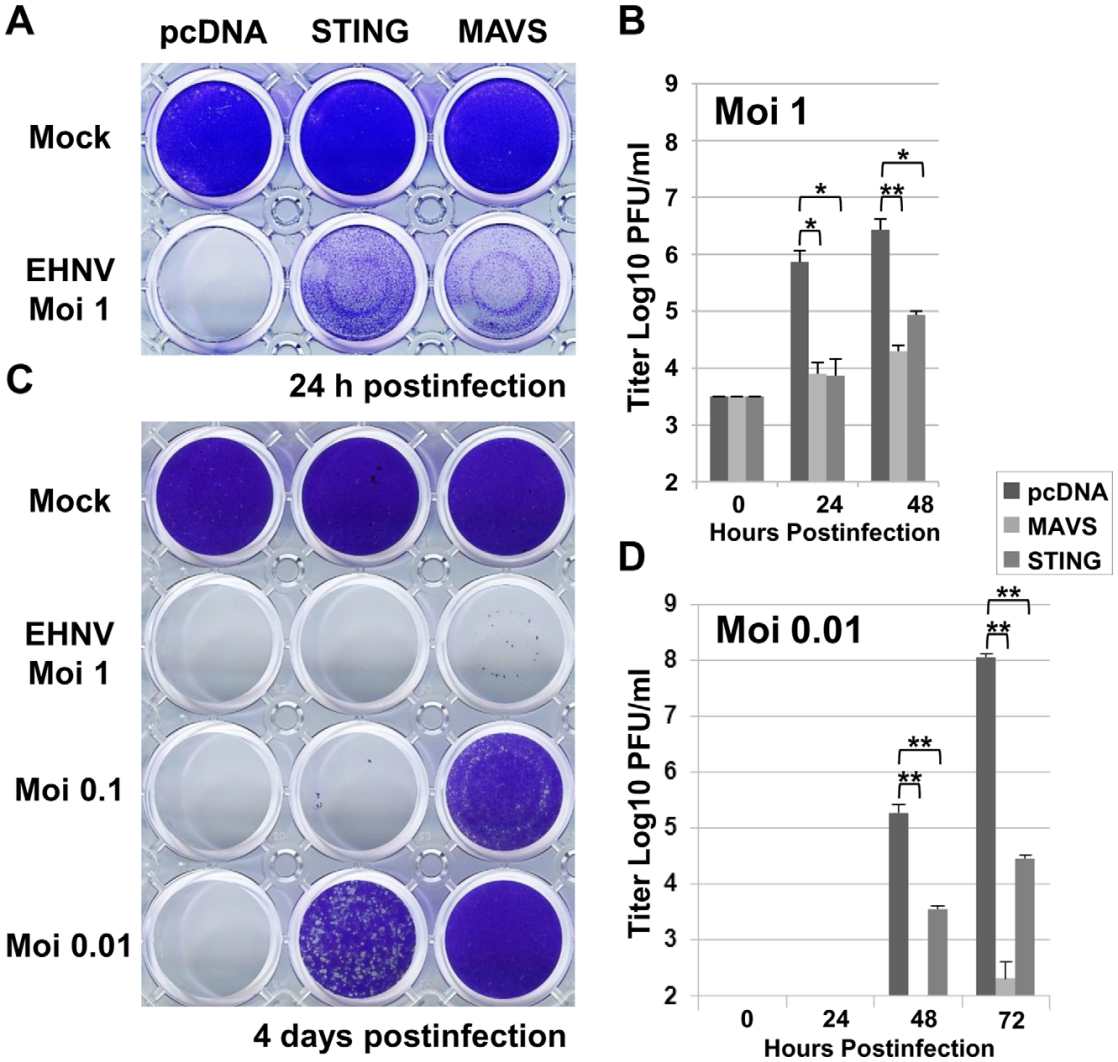
Overexpression of STING induces an antiviral immunity against a DNA virus. EPC cells were transfected with 2 µg of pcDNA-STING, and pcDNA-MAVS or an empty vector (pcDNA) as positive and negative controls, respectively. At 48 h posttransfection, EPC cells were infected with a DNA virus of the Iridoviridae family, i.e., EHNV, at MOI of 1, 0.1 and 0.01 (A and C). The culture supernatants were collected at 0, 24, 48 and 72 h postinfection and the viral titer was determined by plaque assay on EPC cells (B and D). Cell monolayers were then stained with crystal violet either at 24 h postinfection (A) or 4 days postinfection (C) depending to the MOI used, as indicated. Each time point was represented by three independent experiments, and each virus titration was done in duplicate. Means are shown together with the standard errors. Asterisks indicate significant difference (*p<0.01 and **p<0.001) as determined by Student’s *t* test.

Since mammalian STING was described to be involved in the induction of the innate immune response against DNA virus [22], we tested whether the protection induced by the overexpression of zebrafish STING was also effective against a double-stranded DNA virus belonging to the Iridoviridae family, the epizootic hematopoietic necrosis virus (EHNV). EPC cells transfected with an empty vector and infected by EHNV at an MOI of 1 led to a complete CPE in 24 hours (Figure 3A). In contrast, the cells overexpressing the zebrafish STING, as well as MAVS, were able to delay the appearance of the total CPE. Although the CPE was almost total, measurement of the viral titer showed that overexpression of STING decreased the viral titer by 100-fold as compared to that in control cells (from 7.4×10^5^ to 7.4×10^3^ PFU/ml at 24 hours postinfection; Figure 3B). The viral production in STING-expressing cells was very low: 2.5-fold increase from the initial titer compared to 321-fold for the control cells. Moreover, this antiviral effect induced by STING overexpression was more evident when the cells were infected at a lower MOI. Indeed, at an MOI of 0.01, the cell monolayer was almost protected for at least 4 days postinfection (Figure 3C) and the final virus titer was reduced by 3,929-fold (Figure 3D). Therefore, STING protein seems to induce a strong anti-viral immunity against both RNA and DNA viruses.

### Overexpression of STING Constitutively Induces Expression of both IFN and IFN-stimulated Genes

**Figure 4:**
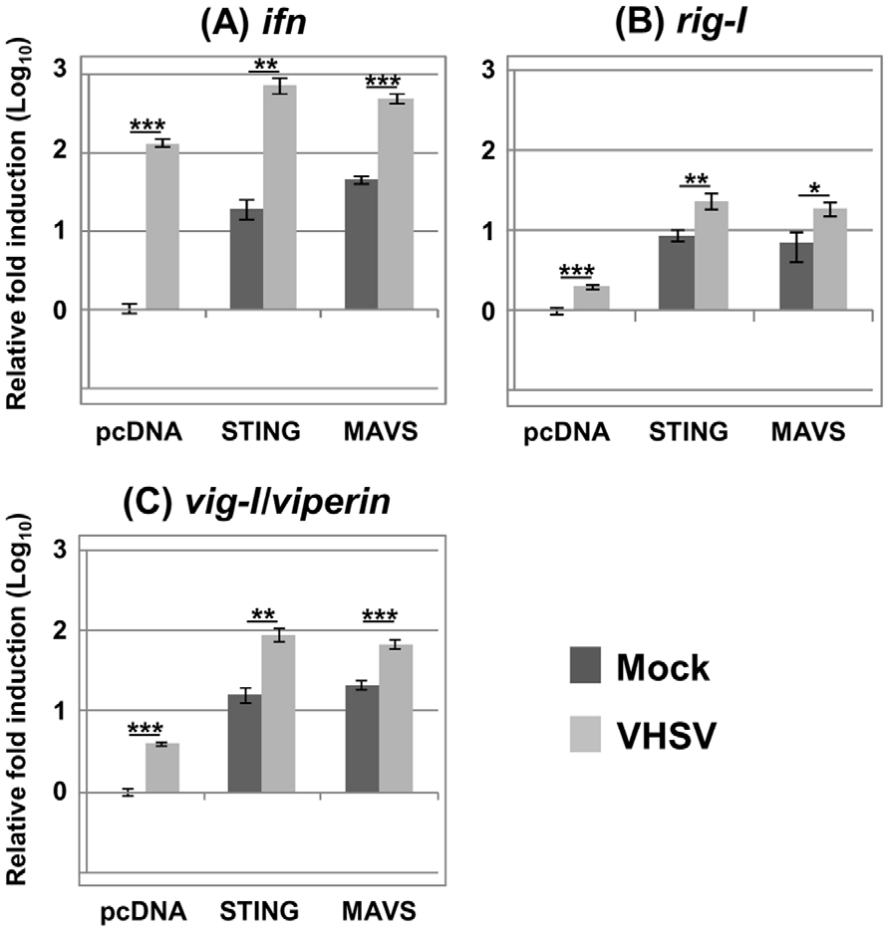
Overexpression of STING induced IFN and ISG expression. EPC cells were transfected with 2 µg of pcDNA vector encoding STING, or MAVS as a positive control, or an empty vector (pcDNA) as a negative control. At 48 h posttransfection, EPC were infected or not with VHSV at an MOI of 1 and incubated at 15°C for 24 h before total RNA extraction. Quantitative Real-time RT-PCR was conducted using primers targeting IFN (A), RIG-I (B) and Viperin (C). The β-actin gene was used as an internal control to normalize the cDNA template and to do real-time PCR calculations. SD of triplicate experiments has been calculated. Asterisks indicate significant difference (*p<0.05, **p<0.01 and ***p<0.001) as determined by Student’s *t* test.

**Figure 5:**
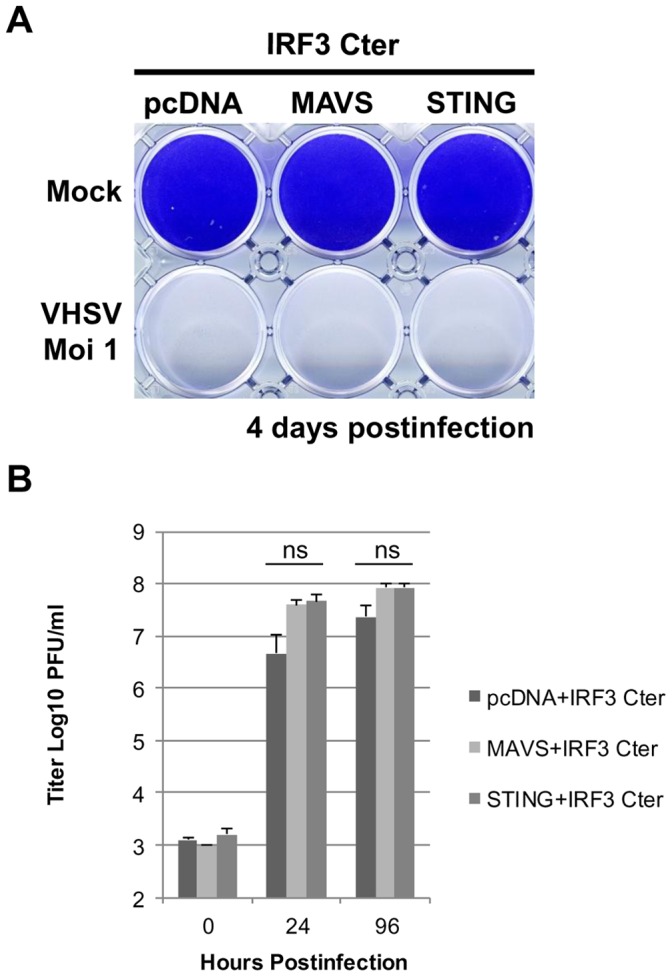
STING antiviral response is mediated by IRF3. EPC cells were co-transfected with 2 µg of pcDNA-IRF3-Cter encoding a dominant-negative mutant of IRF3, and 2 µg of pcDNA-STING, pcDNA-MAVS or an empty vector. At 48 h posttransfection, EPC cells were infected with a recombinant rVHSV-Tom expressing the tdTomato fluorescent protein at an MOI of 1 and incubated at 15°C. The culture supernatants were collected at 0, 24 and 96 h postinfection and the viral titer was determined by plaque assay on EPC cells. Cell monolayers were then stained with crystal violet either at 96 h postinfection. Each time point was represented by three independent experiments, and each virus titration was done in duplicate. Means are shown together with the standard errors. “ns” indicate non-significant difference (p>0.05) as determined by Student’s *t* test.

EPC cells overexpressing zebrafish STING were infected or not by VHSV at an MOI of 1 and total RNA was extracted at 24 hours postinfection. Using real-time RT-PCR, the induced mRNA expression of IFN and IFN-stimulated genes (ISG) was quantified. Results indicated that STING overexpression have a strong effect on the IFN induction with 19-fold mRNA increase compared to the empty vector transfected control (Figure 4A). VHSV infection was also followed by induction of IFN mRNA synthesis (130-fold) and a significant cumulative effect (37-fold) could be observed in cells overexpressing STING and infected with VHSV (Figure 4A). The induction of two previously described IFN-stimulated genes: the fish ortholog of *rig-I* [33] and *vig-1* (virus induced gene 1) the fish ortholog of the mammalian viperin [53], [54] was also examined. As expected, these two genes were induced by VHSV infection at 24 hours postinfection (Figure 4B and C). In cells overexpressing STING, both genes were strongly induced. RIG-I mRNA expression was increased by 9-fold compared to the empty vector transfected control (Figure 4B). Similarly, vig-1 mRNA expression was raised by 16-fold (Figure 4C). Interestingly, in STING-overexpressing cells, the induction of RIG-I and vig-1 was significantly increased by a subsequent VHSV infection by 2.6- and 5.5-fold compared to the mock-infected condition at 24 hours postinfection. Moreover, the induction of the antiviral response by STING overexpression involved the transcription factor IRF3. Indeed, the co-expression of a dominant-negative of IRF3 corresponding to the C-terminal part of the protein (IRF3 Cter; amino acids 115 to 449; GenBank accession no. HE856621) together with STING or MAVS abolished the protection as shown by the apparition of a complete CPE 3 days post-infection (Figure 5A) and a massive virus production (Figure 5B). Thus, overexpression of STING led to a strong induction of both IFN and IFN-stimulated genes which is depending on IRF3 activation.

### Dominant-negative Mutants of MAVS and STING Impairs RIG-I Nter Induced Type I IFN Production

**Figure 6:**
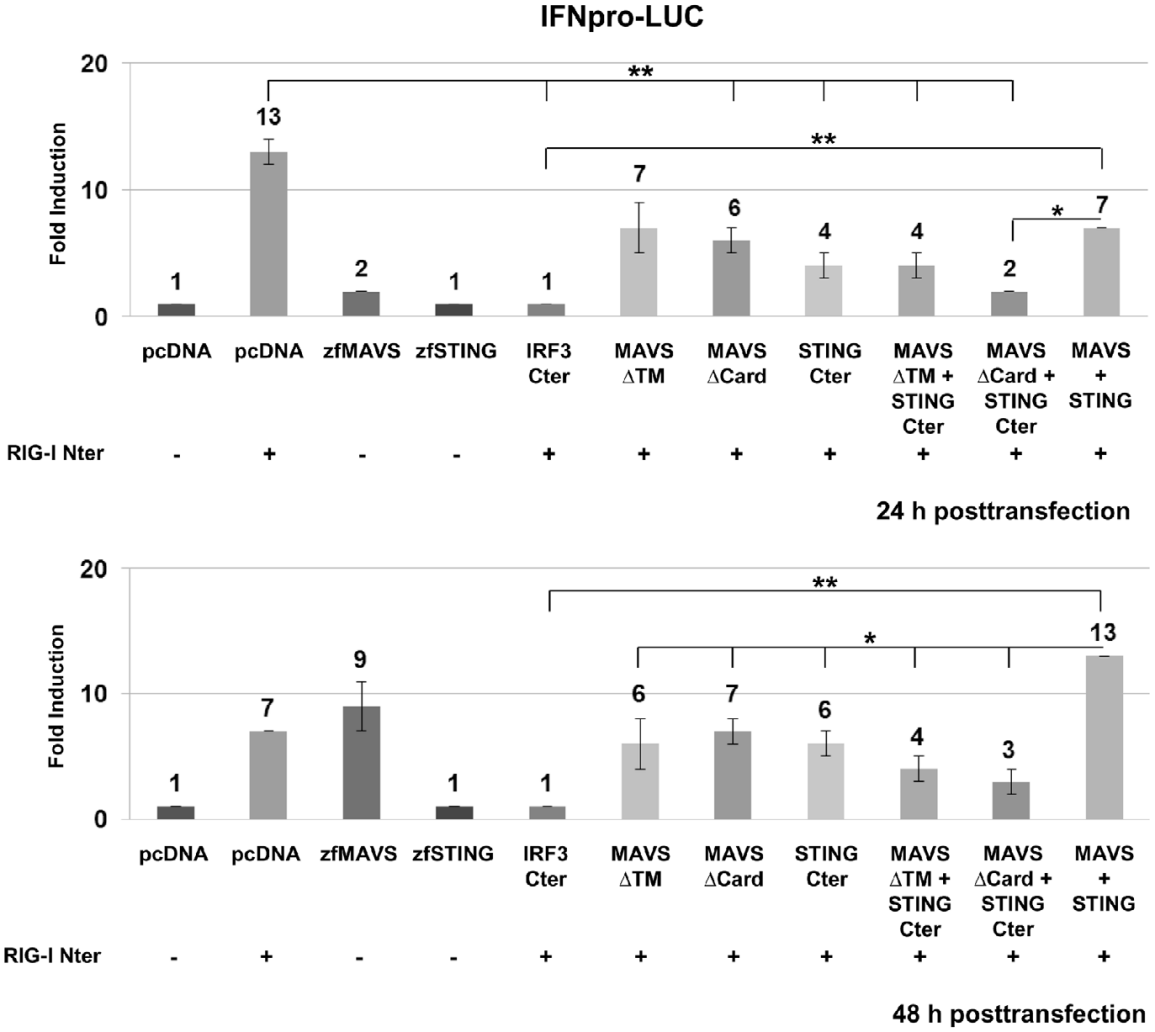
STING and MAVS mediate RIG-I induction of interferon. EPC cells were transfected with 1 µg of pIFNproLUC reporter in combination with various plasmid constructs (1 µg each, except for dominant-negative mutants of MAVS and STING combinations where 0.5 µg of each plasmid was used) as mentioned under each histogram. An empty vector (pcDNA) was added in some experiments to keep the total amount of transfected DNA constant (3 µg total DNA for 5×10^6^ cells). In the condition were pRIG-I Nter-eGFP was not present in the transfection mixture, a peGFP vector was added. At 24 h and 48 h posttransfection, eGFP and luciferase signals were determined. Values of luciferase activities were normalized to the levels of eGFP fluorescence. The fold induction was calculated as the ratio of stimulated versus unstimulated (pcDNA alone) samples. Means of three independent experiments are shown together with the standard errors. Student’s *t*-test was used for statistical analysis, and asterisk indicate significant differences (*p<0.05 and **p<0.01).

RIG-I is the most upstream molecule and IRF3 the most downstream molecule involved in triggering the activation of IFN type-I promoter in response to RNA viruses (see [11] for review), thus it was of interest to examine the implication of MAVS and STING molecules in this pathway. In a previous study we have shown that a constitutively active form of RIG-I (RIG-I Nter) induced IFN secretion and protected EPC cells against VHSV infection ([33] and Figure S1A). To investigate the IFN induction pathway, we constructed an IFN1 reporter plasmid that carries the IFN1 promoter of EPC cells (GenBank accession no. HE856618) driving the expression of a firefly luciferase gene (IFNpro-LUC). This reporter was transfected into EPC cells along with a constitutively expressed eGFP or RIG-I Nter-eGFP reporters to monitor cytotoxicity and transfection efficiency. As expected, overexpression of RIG-I Nter strongly induced the IFN1 promoter (13-fold at 24 h posttransfection) which was not affected by the empty vector control (pcDNA), but was efficiently blocked by IRF3-Cter (Figures 6 and S1B). These results are in accordance with the bioassay data obtained in Figure S1A. Similarly, overexpression of zebrafish MAVS induced the IFN1 promoter, with a peak at 48 h posttransfection (Figure 6). However, despite the demonstration that the overexpression of STING induces both IFN and ISGs, no induction of IFN promoter was observed after zebrafish STING expression in these conditions.

To investigate the involvement of MAVS and STING in RIG-I pathway, we used negative-dominant mutants of MAVS and STING, as previously reported [16], [21] and measured their potential effect on RIG-I Nter constitutive induction of IFN1 promoter. First, the coexpression of full-length MAVS and/or STING together with RIG-I Nter delayed the induction of the IFN1 promoter. Indeed, a similar peak was obtained at 48 h posttransfection instead of 24 h posttransfection with RIG-I Nter alone. In contrast, the negative-dominant mutants of MAVS, MAVSΔCard which lacks the CARD-like domain [33], and STING, STING Cter which corresponds to the 223 C-terminal amino acids, have a significant effect on RIG-I Nter induction of IFN1 promoter by reducing it to 2- and 3-fold, respectively (Figure 6, 24 h posttransfection). The deletion mutant, MAVSΔTM, which is no longer expressed to the outer mitochondrial membrane, also have an inhibitory effect on RIG-Nter but at a lesser extent than that of MAVSΔCard. Interestingly, coexpression of MAVSΔCard together with STING Cter conducts to an almost complete inhibition of IFN1-induction mediated by RIG-I Nter (6.5-fold reduction at 24 h posttransfection). This inhibition is maintained up to 48 h postransfection (3-fold reduction) and significantly relevant compared to the induction observed when full-length MAVS and STING were cotransfected with RIG-I Nter (3.5- and 4.3-fold reduction at 24 h and 48 h posttransfection, respectively). Therefore, MAVS and STING are both downstream signaling molecules of RIG-I.

### The STING and MAVS Proteins Closely Localize in Mitochondrial-ER Contact Regions

**Figure 7:**
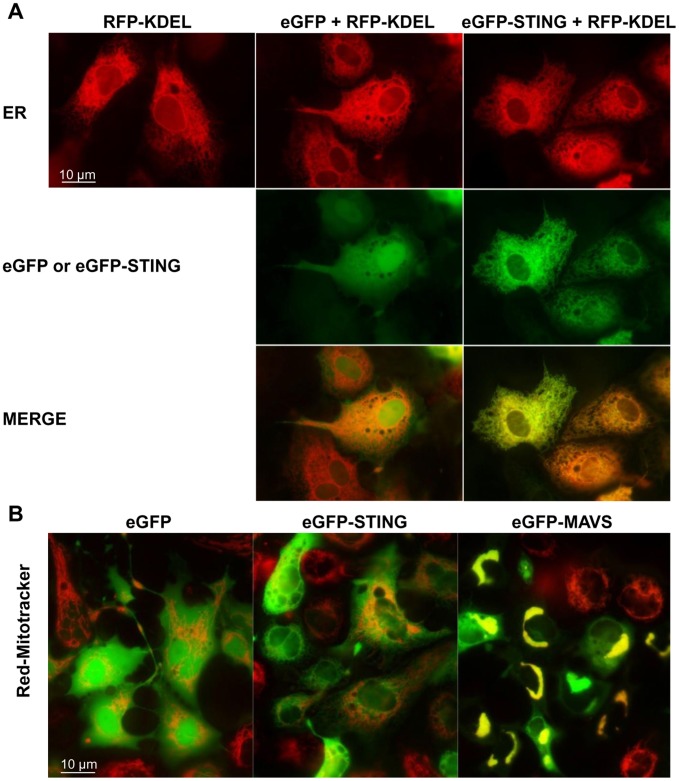
Localization of zebrafish STING to endoplasmic reticulum. peGFP-STING or peGFP-MAVS and peGFP, as negative controls, were transfected together with a plasmid encoding the Red Fluorescent protein fused to a reticulum endoplasmic location signal (RFP-KDEL) into EPC cells (A). The mitochondria were *in vivo* stained with a red MitoTracker (B). The cells were imaged by microscopy 24 h post-transfection. The yellow staining in the overlay image indicates colocalization of STING and RFP-KDEL (A) or MAVS and MitoTracker (B).

**Figure 8:**
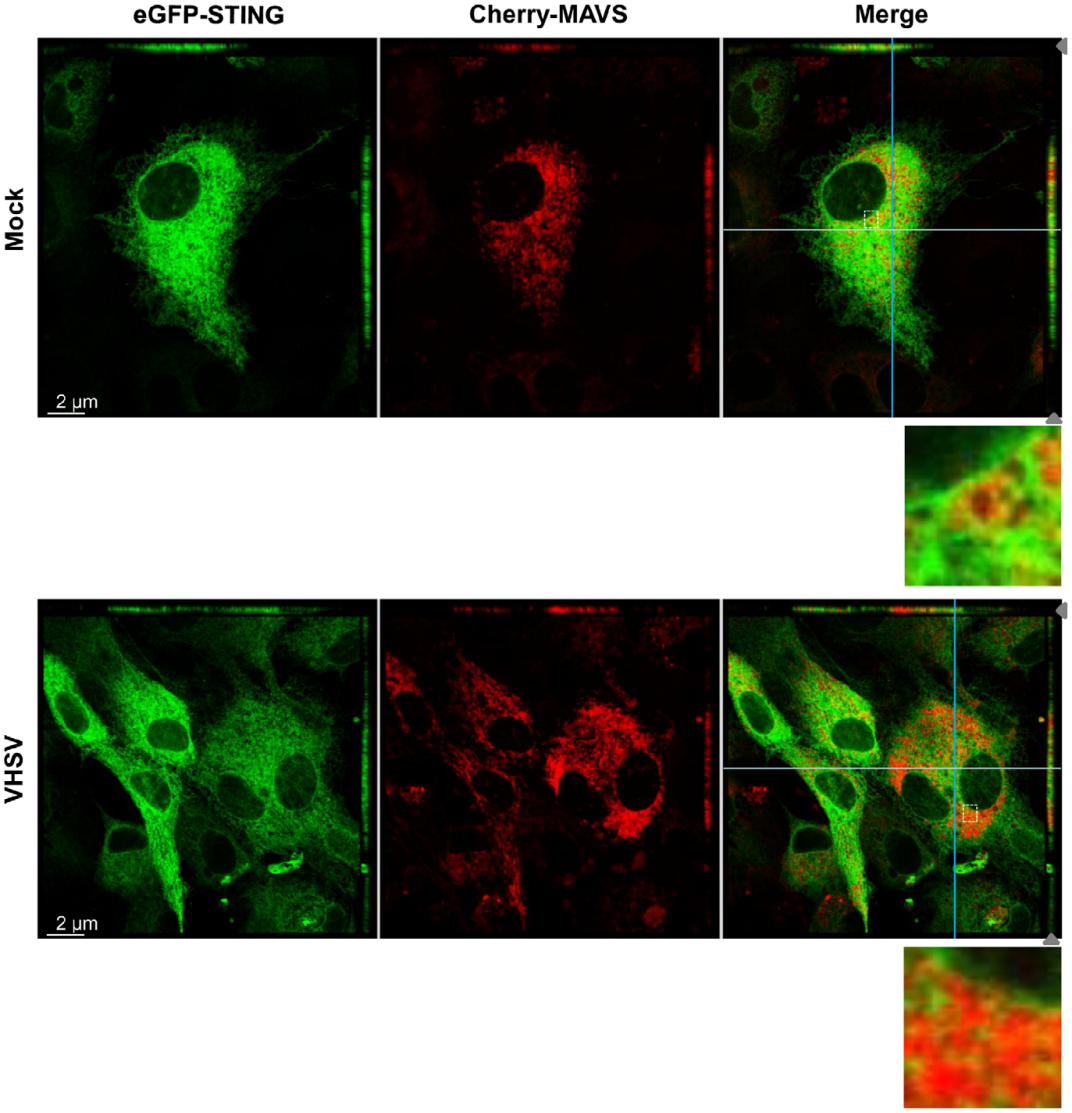
STING and MAVS are in close vicinity in mitochondrial-ER contact regions. EPC cells were cotransfected with 1 µg of peGFP-STING and 1 µg of pCherry-MAVS. At 48 h posttransfection, EPC were infected with VHSV at an MOI of 1 and incubated at 15°C for 24 h before imaging by confocal microscopy. For both panels the main images show a section of the cell monolayer in the *xy* plane at the *z* position indicated by the grey arrow head in the *xz* plane (small top panel) and the *yz* plane (small right side panel). Orthogonal projections of confocal sections shown in the top and right side panels are in the cutting plane indicated by the white and the blue lines corresponding to the *xz* and *yz* planes, respectively. Zoomed insets of boxed areas in merged images are also presented.

Structural analysis of the human STING revealed that the protein contains four hydrophobic transmembrane domains and is inserted in the membrane of the ER [21], [52]. Four transmembrane domains and several ER retention signals (RXR) were also predicted in the fish STING (Figure 1A), suggesting that these proteins may also be targeted to the ER. To determine the subcellular localization of zebrafish STING, EPC cells were transfected with an expression vector, peGFP-STING, encoding a fusion protein eGFP-STING. Since it was described that human STING tagged at either its amino- or carboxy-termini with GFP did not retain its activity [21], we first control whether a zebrafish eGFP-STING fusion protein was still active. In fact, transfection of EPC cells with eGFP-STING vector was still able to confer to EPC cells protection against VHSV infection at a somewhat similar level compared to that induced by STING (Figure S2A and B). Fluorescent microscopy on living cells expressing eGFP-STING and RFP-KDEL, a fluorescent marker that accumulates specifically in the ER, showed a pattern of zebrafish STING localization that is superposed with the ER (Figure 7A). In contrast, cells transfected with the peGFP vector showed a cytosolic and nuclear localization of the GFP protein that does not overlap with RFP-KDEL staining (Figure 7A). Since human STING was also described localizing to the outer membrane of mitochondria [25], fluorescent microscopy was performed on living cells expressing eGFP-STING and stained with MitoTracker, a fluorescent marker that accumulates specifically into the mitochondria (Figure 7B). The pattern of eGFP-STING localization was clearly not overlapping with the MitoTracker staining, as observed with eGFP alone. In contrast, cells transfected with the peGFP-MAVS vector showed a pattern that superposes with MitoTracker staining (Figure 7B), as previously described [33]. Since overexpression of eGFP-STING or Cherry-MAVS was sufficient to induce IFN1 production, it was of interest to see whether these two proteins could colocalize upon activation of the signaling cascade. As previously observed [55], [56], Cherry-MAVS overexpression leaded to the aggregation of the mitochondrial network (Figure 7B and 8). This aggregation was enhanced by VHSV infection. In EPC cells coexpressing eGFP-STING and Cherry-MAVS, both proteins were slightly stained together except for some areas where an overlapping staining could be observed (Figure 8, zoomed area). This overlapping staining was significantly increased by VHSV infection. Thus, STING seems to be localized to the ER in close vicinity to the mitochondrial MAVS protein.

## Discussion

In this article, we report the identification and functional characterization of the STING protein from two fish species (zebrafish and fathead minnow), as a potential key cellular factor for innate immune defence against viruses. The fish *sting* gene encodes a protein with a conserved structure among vertebrates containing four N-terminal transmembrane domains and a cytosolic C-terminal domain. In mammals, the C-terminal domain was shown to function as a dimer in which the highly conserved region between all vertebrates and previously predicted as a TM domain (Figure 1A; TM5) serves as a hydrophobic dimer interface [52]. Mutational analysis of this domain demonstrated that dimerization of STING is essential for induction of IFN [24], [52]. Interestingly, the isoleucine in position 199 of mouse STING is also conserved between vertebrates (Figure 1A). The mutation of this isoleucine to an asparagine (I199N) was previously reported as the basis for the type I IFN expression defect in response to *listeria* infection in a mouse model called Goldenticket [29]. Other residues like S162, Y167, and T263 found in human STING and shown to be critical for binding c-di-GMP are also highly conserved between fish and mammals [52]. The fish STING proteins were also found on the ER membrane, confirming that it shares this location with its mammalian counterpart. Moreover, phylogenetic and synteny analysis indicate that fish STING are true orthologs of the mammalian STING. Finally, this work demonstrates that overexpression of fish STING is sufficient to induce a high expression of both IFN and ISGs, such as the fish orthologs of mammalian RIG-I and viperin. More interestingly, overexpression of these STING proteins in fish cell has a strong antiviral effect on infection by both RNA and DNA viruses (rhabdovirus and iridovirus, respectively) as previously observed in mammalian models [21], [22], [24], [25]. Altogether these indications firmly establish that a *sting* gene encoding an ER protein involved in the innate immune response was already expressed by the common ancestor of fish and mammals.

STING has been described as a key component for an efficient and early induction of type I IFN and required for protection against infection with the negative-stranded virus VSV [21], [22], [25]. However, STING-deficient cells still remain partially able to produce some type I IFN in response to infection with Sendai virus and VSV [21]. It was suggested that STING functions to selectively enhance the RIG-I and MAVS pathway by acting downstream or in parallel of MAVS and facilitating the recruitment of TBK1 and the subsequent phosphorylation of IRF3. Indeed, STING is able to bind RIG-I complexes but it is unclear whether STING directly interacts with MAVS or as a complex with RIG-I. The fact that loss of STING had no significant effect on synthetic dsRNA (poly IC) signaling which is largely mediated by MDA5, another partner of MAVS, suggests that STING may associate with RIG-I-MAVS complexes but not with that involving MDA5-MAVS. Thus, STING is a key scaffolding protein that links RIG-I, rather than MDA5, to MAVS. STING localizes to the ER membrane. The ER is tightly juxtaposed to mitochondria in areas termed mitochondria-associated membranes (MAM), thereby making STING and MAVS in a close vicinity. Recently, it has been shown that MAM is the major site of MAVS signaling [57]. Indeed, during RNA virus infection, RIG-I is recruited to the MAM to bind MAVS and STING. Thus MAM are important areas that mediate formation of intracellular immune platform to recruit downstream molecules and direct antiviral innate immunity. In the present study, we showed that zebrafish eGFP-STING fusion protein, that retains its activity, was expressed at the ER membrane. Forced expression of MAVS and Cherry-MAVS leads to a constitutive signaling and the aggregation of the mitochondrial network. This was not observed after STING overexpression alone although a constitutive signaling was activated. This redistribution of the mitochondrial network has an effect on MAVS and STING co-distribution and is enhanced by VHSV infection. This is correlated with the significant increase of IFN and ISGs mRNA synthesis in VHSV-infected cells compared to mock-infected cells. Recent studies have shown that mitofusin 1 (MFN1), a regulator of the mitochondrial fusion machinery, was associated with MAVS and positively regulated RIG-I pathway, suggesting that mitochondrial dynamics was important for mitochondria-ER association required for innate antiviral responses [56], [58]. Thus, as observed in mammals, fish mitochondria and ER play an important function in the antiviral innate immunity by creating a platform where MAVS and STING transduce the signal to downstream molecules. Moreover, coexpression of dominant-negative mutants of MAVS and STING (MAVSΔCard and STING Cter, respectively) conducts to an almost complete inhibition of the constitutive IFN1-induction mediated by RIG-I Nter, demonstrating that MAVS and STING are both important signaling molecules of RIG-I. Separately, both mutants significantly reduce IFN1-promoter induction, but not completely suggesting that STING and MAVS may represent redundancy signaling molecules of RIG-I. However, the endogen expression of the full-length molecules can explain the remaining induction. Additional experiments will be needed to characterize the sequential events of the activation cascade and the interaction involved between all these molecules. It is unclear whether STING and MAVS can independently transduce the RIG-I signal or need to interact together or via the RIG-I-MAVS complex to be activated and recruit TBK1 and IRF3. In a recent study, Sun and colleagues demonstrated that STING from goldfish interacts in a complex together with TBK1 and IRF3, but did not investigated the presence of MAVS in this complex [38].

The sensing of pathogen-associated DNA in the cytoplasm to trigger host defense is of major interest since several DNA pathogens represent serious threats for aquaculture development. Interestingly, STING has been demonstrated to be critical for the induction of type I IFN by DNA pathogen. Indeed, STING-deficient mice failed to produce type I IFN in response to infection with herpes simplex 1 (HSV1) or intracellular bacteria such as *Listeria monocytogenes* [21], [22]. Although STING was shown to be able to directly bind cyclic di-GMP [52], [59], a product release by bacteria such as *Listeria monocytogenes*, it is clear that STING requires upstream DNA sensors. Recent studies in mammals have describes multiple DNA sensors contributing to an antiviral DNA recognition response, leading to TBK1-dependent IRF3 phosphorylation via STING activation, including the DNA binding protein DAI, the interferon-inducible sensor IFI16 and the helicase DDX41 [60], [61], [62]. In the present study, STING activation by forced expression was shown to be protective against DNA virus infection by both delaying the apparition of total CPE and reduction of the viral production. Thus, STING could also be involved in early detection of pathogen-associated DNA in fish cell cytoplasm. To date, no DNA sensors have been described in teleost fish. Finally, STING-deficient mice immunized with plasmid DNA encoding the ovalbumin gene showed considerably less OVA-specific IgG, as well as reduced IFN γ secretion compared to control mice. Thus, STING is required for both an effective innate and adaptive immune response. Better knowledge on STING pathway could lead to the development of new efficient adjuvant that stimulate the innate and specific immune responses and avoid unwanted inflammatory side effects. This is of interest to aquaculture where DNA vaccines against Novirhabdoviruses, such as VHSV, were found protective after injection in fish [63]. Additional knowledge about these various DNA sensors and redundant DNA sensing pathways throughout the vertebrate evolution will help unravel this complex system for countering pathogen invasion.

In conclusion, this study demonstrates that orthologs of mammalian STING constitute potential key components of an RLR pathway leading to IFN and ISGs production and antiviral immunity against RNA and DNA viruses in teleost fish. These data strongly support that a functional RLR pathway is conserved in vertebrates in which MAVS and STING play a central role.

## Supporting Information

Figure S1 **Induction of IFN promoter by a constitutively active form of RIG-I (RIG-I Nter) is mediated by IRF3.** (A) EPC cells were transfected with 2 µg of pRIG-I Nter-eGFP vector encoding RIG-I Nter fused to the N-terminal end of eGFP in combination with an empty vector (pcDNA) or a pcDNA-IRF3 Cter encoding a dominant-negative mutant of IRF3. At 24 h posttransfection, EPC were infected with rVHSV-Tom at an MOI of 1 and then incubated at 15°C. 48 h hours postinfection, cell monolayers were visualized under a UV-visible light microscope. The viral titer was determined from each culture supernatant by plaque assay. (B) EPC cells were transfected with 1 µg of pIFNproLUC reporter in combination with various plasmid constructs (1 µg each) as indicated under each histogram. An empty vector (pcDNA) was added in some experiments to keep the total amount of transfected DNA constant (3 µg total DNA for 5×10^6^ cells). In the condition were pRIG-I Nter-eGFP was not present in the transfection mixture, a peGFP vector was added. At 24 h posttransfection, eGFP and luciferase signals were determined. Values of luciferase activities were normalized to the levels of eGFP fluorescence. The fold induction was calculated as the ratio of stimulated versus unstimulated samples. Means of four independent experiments are shown together with the standard errors. Asterisks indicate significant difference (*p<0.01) as determined by Student’s *t* test.
(TIF)

Figure S2 **The eGFP-STING fusion protein is fully active.** EPC cells were transfected with 2 µg of peGFP-STING encoding STING fused to the C-terminal end of eGFP or a peGFP vector as a control. At 48 h posttransfection, EPC were infected with rVHSV-Tom at an MOI of 1 and then incubated at 15°C. The nuclei were stained in vivo with Hoeschst (blue) and cell monolayers were visualized under a UV-visible light microscope at 24 h postinfection. The viral titer was determined from each culture supernatant by plaque assay at 0, 24 and 96 h postinfection (B). Each time point was represented by three independent experiments, and each virus titration was done in duplicate. Means are shown. Asterisks indicate significant difference (*p<0.01) and “ns” non-significant difference as determined by Student’s *t* test.
(TIF)

Table S1 Description of the plasmid constructs used in this study.
(DOCX)
