# Supplementary figures and images for: Both STING and MAVS Fish Orthologs Contribute to the Induction of Interferon Mediated by RIG-I
Source: PLoS One. 2012 Oct 16;7(10):e47737. doi: 10.1371/journal.pone.0047737 (PMC3473018; doi:10.1371/journal.pone.0047737)

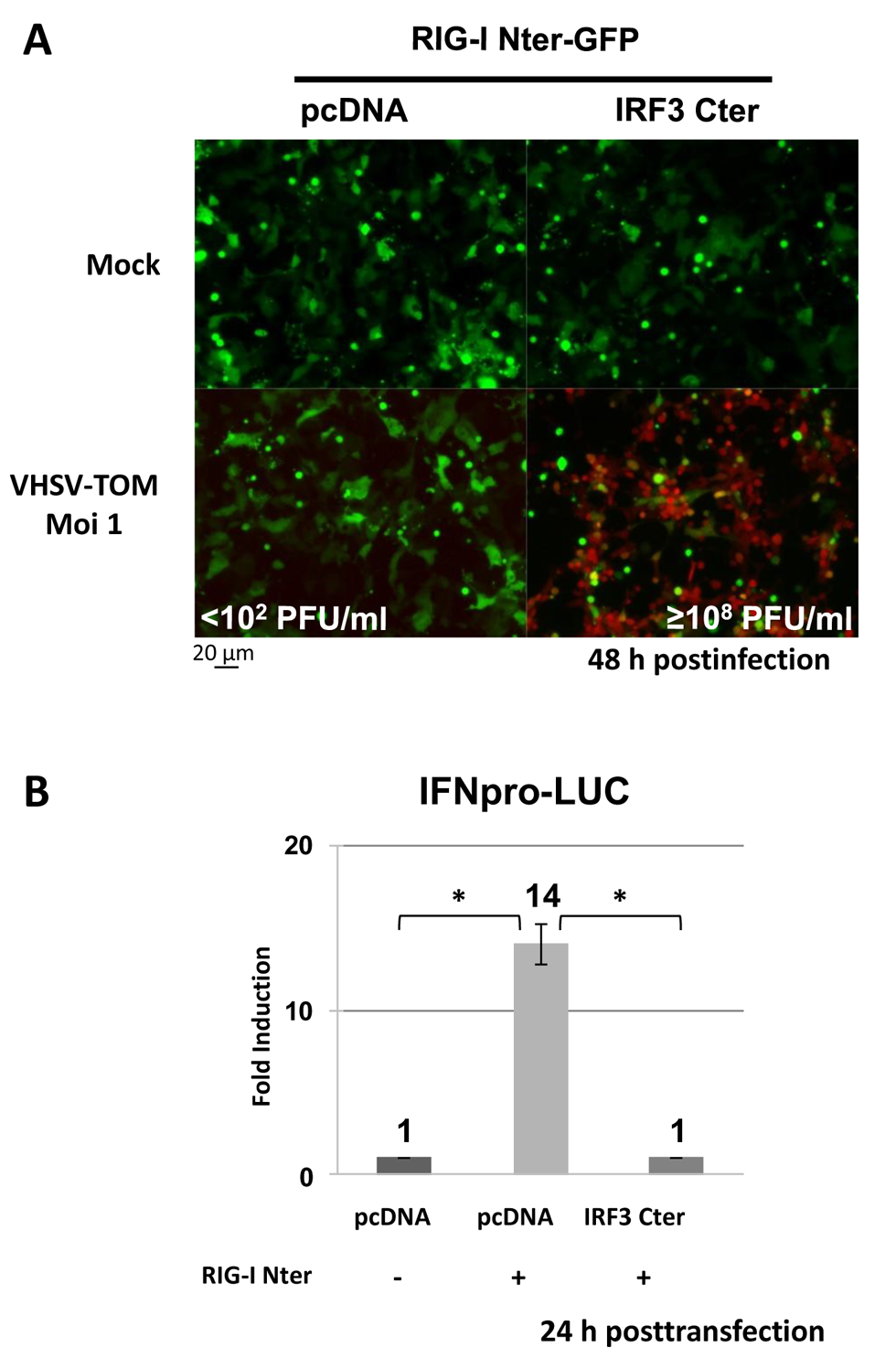

Supplement: Figure S1 — Induction of IFN promoter by a constitutively active form of RIG-I (RIG-I Nter) is mediated by IRF3. (A) EPC cells were transfected with 2 µg of pRIG-I Nter-eGFP vector encoding RIG-I Nter fused to the N-terminal end of eGFP in combination with an empty vector (pcDNA) or a pcDNA-IRF3 Cter encoding a dominant-negative mutant of IRF3. At 24 h posttransfection, EPC were infected with rVHSV-Tom at an MOI of 1 and then incubated at 15°C. 48 h hours postinfection, cell monolayers were visualized under a UV-visible light microscope. The viral titer was determined from each culture supernatant by plaque assay. (B) EPC cells were transfected with 1 µg of pIFNproLUC reporter in combination with various plasmid constructs (1 µg each) as indicated under each histogram. An empty vector (pcDNA) was added in some experiments to keep the total amount of transfected DNA constant (3 µg total DNA for 5×10^6^ cells). In the condition were pRIG-I Nter-eGFP was not present in the transfection mixture, a peGFP vector was added. At 24 h posttransfection, eGFP and luciferase signals were determined. Values of luciferase activities were normalized to the levels of eGFP fluorescence. The fold induction was calculated as the ratio of stimulated versus unstimulated samples. Means of four independent experiments are shown together with the standard errors. Asterisks indicate significant difference (*p<0.01) as determined by Student’s t test. (TIF) [file pone.0047737.s001.tif]

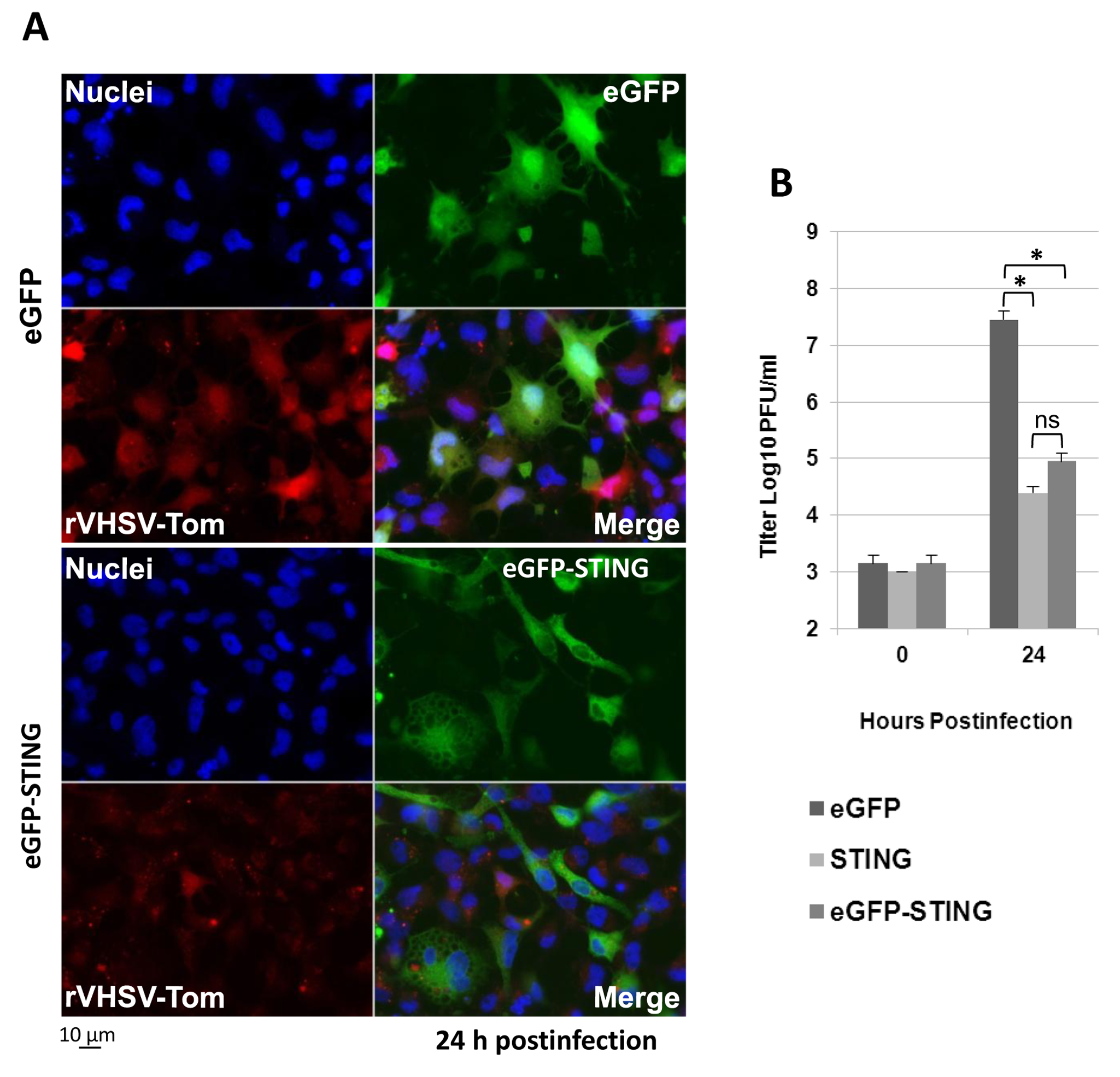

Supplement: Figure S2 — The eGFP-STING fusion protein is fully active. EPC cells were transfected with 2 µg of peGFP-STING encoding STING fused to the C-terminal end of eGFP or a peGFP vector as a control. At 48 h posttransfection, EPC were infected with rVHSV-Tom at an MOI of 1 and then incubated at 15°C. The nuclei were stained in vivo with Hoeschst (blue) and cell monolayers were visualized under a UV-visible light microscope at 24 h postinfection. The viral titer was determined from each culture supernatant by plaque assay at 0, 24 and 96 h postinfection (B). Each time point was represented by three independent experiments, and each virus titration was done in duplicate. Means are shown. Asterisks indicate significant difference (*p<0.01) and “ns” non-significant difference as determined by Student’s t test. (TIF) [file pone.0047737.s002.tif]
