# Supplementary material for: Both STING and MAVS Fish Orthologs Contribute to the Induction of Interferon Mediated by RIG-I
Source: PLoS One. 2012 Oct 16;7(10):e47737. doi: 10.1371/journal.pone.0047737 (PMC3473018; doi:10.1371/journal.pone.0047737)
Supplement: Table S1 — Description of the plasmid constructs used in this study. (DOCX) [file pone.0047737.s003.docx]

**Supplemental Table 1**: **Description of the plasmid constructs used in this study**.

| **Plasmid name** | **Product** | **Brief description** |
| --- | --- | --- |
| pcDNA-STING | STING | Full length STING molecule from zebrafish or epc cells |
| pcDNA-STING Cter | STING Cter | C-terminal domain of STING (aa 177 to 398) |
| pcDNA-MAVS | MAVS | Full length MAVS molecule from zebrafish [33] |
| pcDNA-MAVS ∆CARD | MAVS ∆CARD | C-terminal domain of MAVS (aa 94 to 606) without the CARD domain [33] |
| pcDNA-MAVS ∆TM | MAVS ∆TM | N-terminal domain of MAVS (aa 1 to 567) without the transmembrane region [33] |
| pcDNA-IRF3 Cter | IRF3 Cter | C-terminal domain of epc IRF3 (aa 115 to 449) without the DNA binding domain |
| pRIG-I Nter-eGFP | RIG-I Nter | N-terminal CARD domain of RIG-I (first 275 aa) fused to eGFP [33] |
| pIFNpro-LUC | Luciferase | LUC gene under the transcriptional control of the IFN1 promoter |
| pcDNA | _ | Empty vector (Invitrogen) |
| peGFP C1 | eGFP | Commercial plasmid (Clontech) |
| peGFP-STING | eGFP-STING | Full length STING molecule fused to the C-terminus of eGFP |
| peGFP-MAVS | eGFP-MAVS | Full length MAVS molecule fused to the C-terminus of eGFP |
| pCherry-MAVS | Cherry-MAVS | Full length MAVS molecule fused to the C-terminus of Cherry |
